# Effects of Selected Plant-Derived By-Products as Partial Soybean Meal Replacements on Nutrient Utilization, Cecal Microbiota, and Performance of Aged Laying Hens

**DOI:** 10.3390/ani16213434

**Published:** 2026-10-30

**Authors:** Qingyu Zhao, Wanling He, Fumiao Sun, Fengyan Wang, Jing Zhang, Zhaoyang Wu, Jiangtao Liu, Zhibin Zhang, Xiaoli Li

**Affiliations:** 1 College of Animal Science and Technology, Henan University of Science and Technology, No. 263 Kaiyuan Road, Luoyang 471000, China; zhaoqingyu@stu.haust.edu.cn (Q.Z.); hwling921@126.com (W.H.); sunfumiao@stu.haust.edu.cn (F.S.); 250320181176@stu.haust.edu.cn (J.Z.); wzy@stu.haust.edu.cn (Z.W.); 15515258516@163.com (J.L.); m15315545345_1@163.com (Z.Z.); 2 Henan Egdoo Technology Group Co., Ltd., No. 221 Lianhua Street, Zhengzhou 450001, China; wangfy12102026@163.com

**Keywords:** aged laying hens, soybean meal substitution, plant-derived by-products, digestive enzyme activities, cecal microbiota, apparent nutrient utilization, productive performance

## Abstract

Soybean meal (SBM) is a major protein source in commercial layer diets; however, fluctuations in SBM prices and supply have increased the need for alternative protein sources in poultry production. Plant processing by-products are considered promising feed resources because of their abundant availability and nutritional value. However, the effects of partially replacing SBM with different plant-derived by-products on digestive function, gut microbiota, nutrient utilization, productive performance, and egg quality in aged laying hens remain insufficiently characterized. Therefore, this study aimed to evaluate the effects of partially replacing SBM with different plant-derived by-products on gastrointestinal digestive enzyme activities, cecal microbial community composition, apparent nutrient utilization, productive performance, and egg quality in aged laying hens. A total of 270 Hy-Line Brown laying hens at 80 weeks of age were randomly assigned to five dietary treatments, with six replicates per treatment and nine hens per replicate. Digestive enzyme activities and cecal microbiota were assessed using three birds per treatment. The control group was fed a corn-SBM basal diet, whereas the experimental groups were fed diets in which SBM was partially replaced with 10% palm kernel meak (PKM, T1), 12% rice bran meal (RBM, T2), 15% sunflower meal (SFM, T3), or 8% *Zanthoxylum bungeanum* seeds (ZBS, T4), respectively. The results showed that digestive enzyme activities varied among dietary treatments, with responses depending on the type of enzyme and gastrointestinal segment examined. Cecal microbiota analysis revealed no significant differences in alpha diversity among dietary treatments (*p* > 0.05), whereas overall microbial community composition differed significantly (PERMANOVA, *p* = 0.015). Differences in the relative abundances of individual bacterial taxa were also observed, but none remained significant after multiple-testing correction. Furthermore, the T3 group exhibited significantly higher apparent utilization of all six measured nutrients than the control group, whereas the other treatments showed mixed, nutrient-specific responses. Dietary treatments affected laying rate and eggshell thickness (*p* < 0.05), whereas average egg weight, average daily feed intake, feed-to-egg ratio, and other measured egg quality parameters were not significantly affected (*p* > 0.05). Overall, the four plant-derived by-products produced variable responses across the measured outcomes, suggesting ingredient-specific potential as partial substitutes for SBM under the dietary formulations and inclusion levels evaluated. However, their equivalent nutritional suitability cannot be established from the present findings, and further studies with larger sample sizes and multiple inclusion levels are warranted.

## Simple Summary

Soybean meal is the primary protein source used in diets for laying hens, but its rising cost and supply uncertainty have increased interest in alternative protein ingredients. In this study, four plant-derived by-products, palm kernel meal, rice bran meal, sunflower meal, and *Zanthoxylum bungeanum* seeds, were evaluated at different inclusion levels as partial substitutes for soybean meal in diets for 80-week-old laying hens. Their effects on digestive enzyme activities, cecal microbiota, apparent nutrient utilization, productive performance, and egg quality were investigated. The results showed that the dietary treatments affected digestive enzyme activities differently across intestinal segments. The overall composition of intestinal bacteria also differed among dietary treatments. Dietary treatments significantly affected laying rate and eggshell thickness, whereas most other productive performance and egg quality parameters were not significantly altered. The sunflower meal diet showed higher apparent nutrient utilization than the control diet, whereas some other treatments reduced the apparent utilization of certain nutrients. Overall, the four plant-derived by-products showed potential as partial replacements for soybean meal under the specific dietary formulations and inclusion levels evaluated in this study. However, these findings do not establish nutritional equivalence among the four ingredients. These findings provide useful information for evaluating plant-derived feed resources and developing diets with reduced soybean meal use for aged laying hens.

## 1. Introduction

Owing to their substantial nutritional value, superior protein quality, and consistent availability, eggs constitute an important component of the global animal-based food system. Consequently, the egg-laying industry holds a pivotal position within contemporary livestock production systems. Advances in modern breeding techniques and management practices for egg-laying chickens have led to an extension of the production cycle for commercial laying hens, with older hens increasingly becoming a crucial component of the egg-laying production system. Nevertheless, as laying hens age, they may exhibit declines in productive performance, diminished nutrient utilization efficiency, and alterations in egg quality, thereby placing higher demands on dietary nutrient levels and feed ingredient utilization efficiency [1,2]. Soybean meal (SBM) is among the most extensively utilized plant protein sources in poultry production, characterized by its high crude protein content, a relatively balanced essential amino acid profile, and high amino acid digestibility. As a result, it has long been the primary protein source in commercial laying hen diets [3,4]. In recent years, fluctuations in global soybean production, changes in international trade, and rising feed prices have increased the cost and supply uncertainty of SBM, placing greater economic pressure on the laying hen industry [5,6]. Consequently, the development of alternative protein sources that are nutritionally valuable, consistently available, and economically feasible is of paramount importance for fostering the sustainable advancement of the poultry industry [7].

Plant-derived by-products are typically abundant and contain varying levels of proteins, lipids, dietary fiber, and functional bioactive compounds, making them potentially valuable feed resources [8,9]. However, differences in their nutritional composition may lead to distinct effects on digestive function, nutrient utilization, and intestinal microbial communities. Therefore, these by-products should not be regarded as nutritionally interchangeable protein sources, but rather as distinct feed ingredients requiring individual evaluation. Consequently, systematically evaluating their effects at selected dietary inclusion levels is important for assessing their potential as partial SBM substitutes in aged laying hen diets and promoting the efficient utilization of agricultural by-products.

Palm kernel meal (PKM) is a major by-product of palm oil processing and contains considerable amounts of protein. However, its relatively high fiber and non-starch polysaccharide contents may constrain nutrient utilization in poultry [10]. Previous research has shown that PKM can be included in laying hen diets at a level of 10% without adversely affecting productive performance, indicating its potential as an alternative feed ingredient [11]. Rice bran meal (RBM), a by-product of rice processing, contains protein, lipids, dietary fiber, and various bioactive compounds, giving it nutritional characteristics distinct from those of PKM. Previous studies have shown that rice bran can be incorporated into laying hen diets at levels of up to 25%, with 10–15% showing favorable responses in certain productive performance parameters [12]. However, its relatively high fiber content, limited phosphorus availability, and other nutritional characteristics may constrain nutrient utilization, and these factors should therefore be considered when determining dietary inclusion levels [12,13]. Sunflower meal (SFM) is widely available and contains a relatively high level of protein, making it a potential alternative protein ingredient for poultry diets. Previous studies have reported that SFM has favorable protein nutritional value and can be used as a protein source in poultry diets [14,15]. Studies in laying hens have further shown that dietary SFM can be included at levels of 5–10% without adversely affecting productive performance, although its effects on nutrient utilization may vary among nutrients [16]. Unlike the other three by-products, *Zanthoxylum bungeanum* seeds (ZBS) contain appreciable amounts of lipids and other nutrients, making them a nutritionally distinct feed resource. However, direct evidence regarding the use of whole ZBS in poultry diets remains limited. In broiler chickens, *Zanthoxylum bungeanum* seed meal has been evaluated as a partial substitute for soybean meal and was shown to influence growth performance and nutrient utilization [17]. More recently, *Zanthoxylum bungeanum* seed cake and meal have been investigated in laying hens, with dietary supplementation affecting productive performance and cecal microbial composition [18]. Collectively, these findings suggest that these four by-products have potential applications in poultry diets but differ in their nutritional characteristics, supporting the need for ingredient-specific evaluation.

Although previous studies have investigated the dietary application of these plant-derived by-products, research has often focused on individual aspects, such as productive performance or nutrient utilization, whereas integrated assessments of digestive function, nutrient utilization, cecal microbial composition, and productive performance in aged laying hens remain limited. Based on previous studies and the distinct nutritional characteristics of the four ingredients, inclusion levels of 10% PKM, 12% RBM, 15% SFM, and 8% ZBS were selected for evaluation. Given their distinct nutritional characteristics, we expected that partial replacement of SBM with PKM, RBM, SFM, or ZBS at their respective inclusion levels would differentially influence digestive enzyme activities and apparent nutrient utilization. Changes in cecal microbial community composition were also anticipated in response to the different dietary treatments. Despite these potential differences in digestive and microbial responses, the selected dietary formulations were expected to maintain productive performance and egg quality in aged laying hens. Accordingly, this study systematically evaluated digestive enzyme activities, apparent nutrient utilization, cecal microbial composition, productive performance, and egg quality to assess the ingredient-specific potential of these four plant-derived by-products as partial SBM substitutes under the dietary formulations and inclusion levels evaluated.

## 2. Materials and Methods

This study was conducted following approval from the Laboratory Animal Management and Use Committee of Henan University of Science and Technology (Approval No. AW20602202-1-2; approved in October 2025). All animal procedures were carried out in accordance with the institutional guidelines for the care and use of laboratory animals.

### 2.1. Animals and Experimental Design

The study was conducted using 80-week-old Hy-Line Brown laying hens sourced from local commercial poultry farms in Henan Province (Luoyang, China). A total of 270 healthy laying hens with similar initial body weights were randomly assigned to five dietary treatments. Each treatment consisted of six replicates, with nine hens per replicate housed in three cages (three hens per cage). Each replicate served as the experimental unit. The experiment lasted 42 days, including a 7-day adaptation period followed by a 35-day feeding trial.

The control group received a corn–SBM basal diet formulated with reference to the nutritional recommendations provided in the Hy-Line Brown Commercial Layers Management Guide [19]. In the four experimental diets, SBM was partially replaced with 10% PKM (T1 group) [11], 12% RBM (T2 group) [20], 15% SFM (T3 group) [16,21], or 8% ZBS (T4 group) [17]. The inclusion levels of the four plant-derived by-products were determined based on previous studies evaluating their application in laying hen diets. PKM was obtained as a by-product of mechanical oil extraction from oil palm kernels, followed by drying and grinding. RBM was produced from rice bran generated during the milling of brown rice, followed by mechanical oil extraction. SFM was produced from sunflower seeds through dehulling, drying, mechanical oil extraction, and grinding. ZBS were obtained as a by-product of *Zanthoxylum bungeanum* processing and were subsequently dried, sieved, and cleaned to remove impurities, without oil extraction. The diet composition is shown in Table 1, and the nutritional levels are shown in Table 2.

All hens were managed in accordance with the recommendations of the Hy-Line Brown Commercial Layers Management Guide [19]. Birds were reared in conventional cages and had unrestricted access to feed and drinking water throughout the study. The house was well ventilated and maintained under a 16 h light:8 h dark photoperiod, with the environmental temperature ranging from 18 to 21 °C. Feed was offered twice daily at 08:00 and 17:00.

### 2.2. Excreta Collection and Apparent Total-Tract Nutrient Utilization Analysis

Prior to sample collection, excreta collection trays were placed beneath the cages. Total excreta were collected at 24 h intervals for three consecutive days, and daily feed intake was recorded simultaneously. Visible contaminants, including feathers and feed residues, were removed. Following each collection, the excreta were treated with 10% hydrochloric acid to minimize nitrogen loss through ammonia volatilization. Excreta from two replicates were first combined, and the samples collected over three consecutive days were subsequently pooled and thoroughly homogenized while fresh, yielding three pooled samples per treatment (*n* = 3). The samples were then dried at 65 °C to a constant weight, ground to pass through a 40-mesh sieve, homogenized, and stored in airtight containers until analysis.

The concentrations of calcium (Ca), total phosphorus (TP), crude protein (CP), dry matter (DM), ether extract (EE), and crude ash (Ash) were determined according to the procedures described by Zhang et al. [22]. Three pooled excreta samples were analyzed per treatment (*n* = 3), with each sample subjected to a single determination for each nutrient parameter.

Apparent nutrient utilization (%) was calculated as:

Apparent nutrient utilization (%) = [(Nutrient intake − Nutrient excretion)/Nutrient intake] × 100.

### 2.3. Assessment of Digestive Enzyme Activity

On the final day of the experiment, samples were collected 1 h after the morning feeding. Three hens per treatment were randomly selected from three different replicate units, with one hen sampled per replicate unit. Digesta samples were collected from the gizzard, duodenum, and jejunum for digestive enzyme activity determination, while cecal contents were collected for microbial sequencing. The collected samples were placed into sterile 5 mL enzyme-free centrifuge tubes, immediately frozen in liquid nitrogen, and stored at −80 °C until analysis.

Pepsin activity in gizzard digesta and the activities of trypsin, chymotrypsin, lipase, and α-amylase in duodenal and jejunal digesta were determined using commercial assay kits (A080-1-1, A080-2, A080-3-1, A054-2-1, and C016-1-2, respectively; Nanjing Jiancheng Bioengineering Institute, Nanjing, China) according to the manufacturer’s instructions. Total protein concentrations in the sample homogenates were determined using a Coomassie brilliant blue assay kit (A045-2; Nanjing Jiancheng Bioengineering Institute, Nanjing, China). Enzyme activities were calculated using the kit-specific formulas, normalized to the protein concentration of the corresponding sample homogenates, and expressed as U/mg protein. Appropriate blank and control measurements were included, and sample measurements were performed within the applicable assay ranges according to the manufacturer’s instructions.

The sampling time (1 h after feeding) was selected based on preliminary experiments conducted in our laboratory, which indicated that digestive enzyme activities were relatively stable and representative at this time point.

### 2.4. Cecal Microbiota Analysis

Approximately 0.20–0.50 g of cecal digesta collected as described in Section 2.3 was transferred into centrifuge tubes containing lysis buffer and homogenized at 60 Hz using a tissue homogenizer (Tissuelyser-48, Shanghai Jingxin Industrial Development Co., Ltd., Shanghai, China). Total genomic DNA was extracted using the E.Z.N.A.^®^ Soil DNA Kit (Cat. No. M5635-02; Omega Bio-Tek, Norcross, GA, USA) according to the manufacturer’s instructions. DNA concentration and purity were determined with a NanoDrop NC2000 spectrophotometer (Thermo Fisher Scientific, Waltham, MA, USA), while DNA integrity was evaluated by 0.8% agarose gel electrophoresis.

The hypervariable V3–V4 region of the bacterial 16S rRNA gene was amplified using the primer pair 338F (5′-ACTCCTACGGGAGGCAGCA-3′) and 806R (5′-GGACTACHVGGGTWTCTAAT-3′). A DNA extraction blank containing no cecal digesta and a PCR negative control containing no template DNA were included to monitor potential contamination during DNA extraction and PCR amplification, respectively. The PCR products were purified following separation on 2% agarose gels and subsequently used for sequencing library construction with the TruSeq Nano DNA LT Library Preparation Kit (Illumina, San Diego, CA, USA). After library quality assessment and quantification, paired-end sequencing was performed on the Illumina NovaSeq 6000 platform using the NovaSeq 6000 SP Reagent Kit (500 cycles).

Raw sequencing reads were processed using QIIME 2 (version 2024.5) following the standard QIIME 2 workflow. Briefly, raw sequences were demultiplexed using the demux plugin, and primer sequences were removed using the cutadapt plugin. The DADA2 plugin was then used for sequence quality filtering, denoising, paired-end read merging, and chimera removal to generate amplicon sequence variants (ASVs). An ASV abundance table was constructed for downstream analyses.

Representative ASV sequences were taxonomically classified against the Greengenes2 database (version 2022.10) using the classification method implemented in QIIME 2. ASVs with an abundance below 0.001% of the total sequencing reads across all samples were removed, and the resulting abundance table was used for downstream analyses. Alpha diversity indices were calculated using QIIME 2. Beta diversity was evaluated using Bray–Curtis dissimilarity matrices and visualized by principal coordinate analysis (PCoA). Differences in microbial community composition among treatments were assessed using permutational multivariate analysis of variance (PERMANOVA; adonis), while homogeneity of multivariate dispersion was evaluated using PERMDISP (permdisp). Differences in the relative abundances of bacterial taxa among dietary treatments were assessed using the Kruskal–Wallis test, with *p*-values adjusted for multiple comparisons using the Benjamini–Hochberg procedure. Taxonomic compositions at the phylum and genus levels were visualized using R software (version 4.5.0).

### 2.5. Analysis of Productive Performance and Egg Quality

During the experimental period, feed intake, egg production, and total egg weight were recorded daily for each treatment group. Egg production rate, average egg weight, average daily feed intake, and feed-to-egg ratio were calculated daily over the 35-day experimental period. The laying rate (%) was calculated as the number of eggs produced divided by the number of hens and multiplied by 100. Average egg weight (g) was calculated as the total egg weight divided by the total number of eggs laid on that day. Average daily feed intake (g/hen/day) was calculated based on the daily feed consumption and the number of laying hens. The feed-to-egg ratio was calculated as feed consumption divided by total egg mass.

On the day after the experimental period, two eggs were randomly selected from each replicate for egg quality assessment, resulting in 12 eggs per treatment group and 60 eggs in total. The selected eggs were weighed individually, and egg length and width were measured using an electronic digital vernier caliper (Delixi, Wenzhou, Zhejiang, China). The egg shape index (%) was calculated as the short diameter divided by the long diameter × 100. Eggshell strength was measured using an eggshell strength tester (RH-DQ200, Guangzhou Runhu Instrument Co., Ltd., Guangzhou, China). The eggs were then broken, and the yolks were separated from the albumen and shells. An electronic digital thickness gauge (Guanglu, Guilin Guanglu Digital Measurement and Control Co., Ltd., Guilin, China) was used to measure eggshell thickness at the sharp end, equatorial region, and blunt end of each egg, and the mean of the three measurements was used as the eggshell thickness. Yolk color, albumen height, and Haugh units were measured using an egg quality multifunctional analyzer (EMT-7300II, Robotmation Co., Ltd., Tokyo, Japan).

### 2.6. Statistical Analysis

Following the initial organization of the experimental data using Microsoft Excel 2019 (Microsoft Corporation, Redmond, WA, USA), a one-way analysis of variance (ANOVA) was conducted utilizing SPSS version 27.0 (SPSS Inc., Chicago, IL, USA). Duncan’s multiple range test was employed for multiple comparisons between groups. The data are expressed as mean ± standard deviation (SD), with statistical significance established at *p* < 0.05. For cecal microbiota analysis, differences in beta diversity among treatment groups were assessed using permutational multivariate analysis of variance (PERMANOVA) based on Bray–Curtis dissimilarity, while permutational analysis of multivariate dispersions (PERMDISP) was performed to evaluate the homogeneity of within-group dispersion. Differences in the relative abundances of bacterial taxa among treatments were evaluated using the nonparametric Kruskal–Wallis test, with the Benjamini–Hochberg (BH) procedure applied to correct for multiple testing. Adjusted *p*-values < 0.05 were considered statistically significant for taxon-level comparisons.

## 3. Results

### 3.1. Effects of Different Plant-Derived By-Products on Digestive Enzyme Activities in Aged Laying Hens

Table 3 presents the effects of dietary plant-derived by-products on digestive enzyme activities in laying hens. Compared with the control group, the T1 group exhibited significantly higher activities of gizzard pepsin, jejunal trypsin, and jejunal α-amylase (*p* < 0.05). In the duodenum, all four enzyme activities were numerically higher in the T1 group, but the differences were not significant (*p* > 0.05). Jejunal trypsin activity was also significantly higher in the T3 group, whereas jejunal α-amylase activity was significantly higher in the T2 and T3 groups (*p* < 0.05). Conversely, several duodenal enzyme activities were significantly lower in the T2 and T4 groups, whereas the T3 group exhibited significantly lower duodenal α-amylase and jejunal chymotrypsin activities (*p* < 0.05). Overall, digestive enzyme activities showed enzyme- and gastrointestinal segment-specific responses to the dietary treatments.

### 3.2. Effects of Different Plant-Derived By-Products on the Cecal Microbiota of Aged Laying Hens

**Figure 1:**
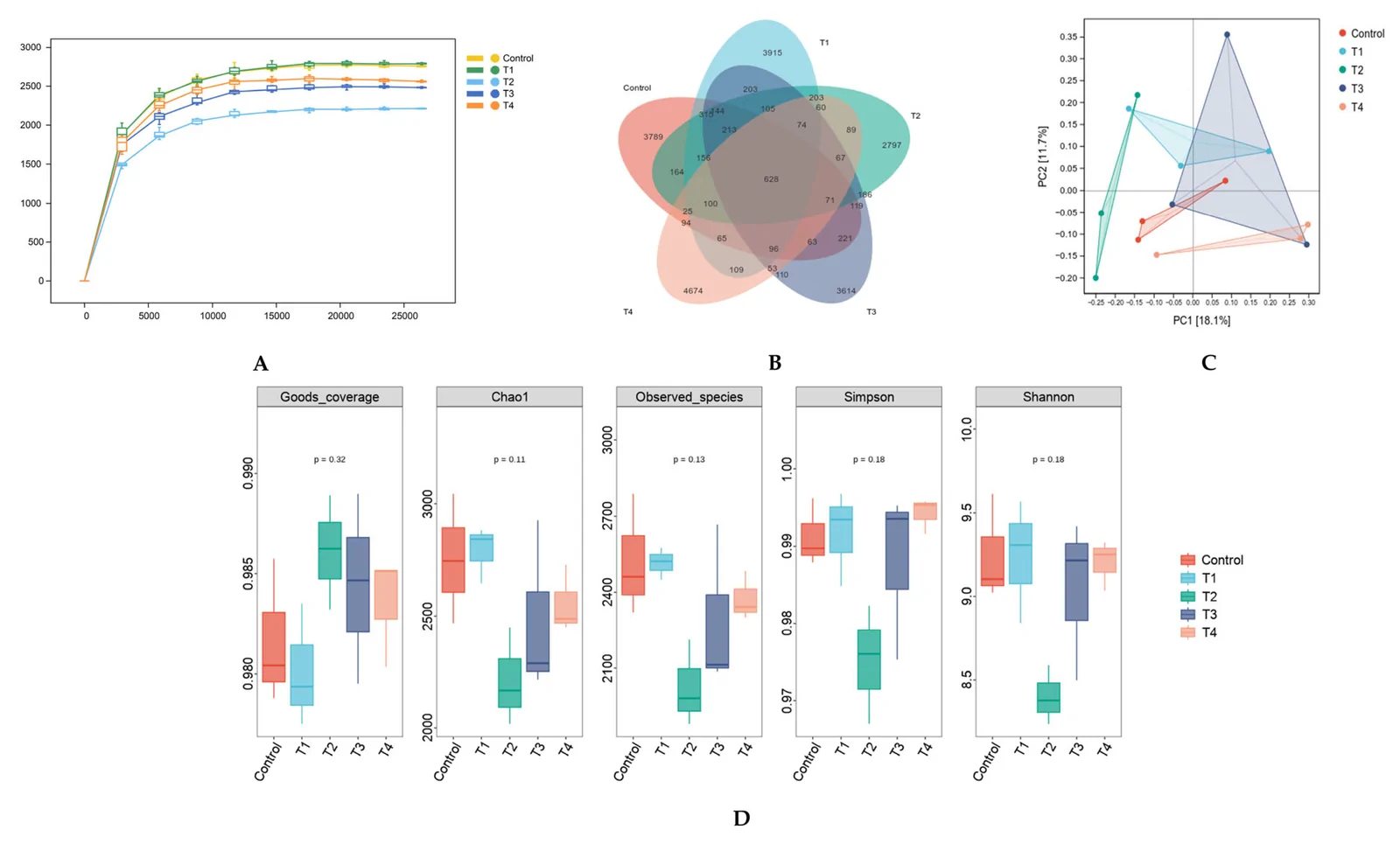
Characterization of the cecal microbiota (*n* = 3). (**A**) Rarefaction curves. (**B**) Distribution of shared and unique ASVs among treatment groups. (**C**) Principal coordinate analysis (PCoA) of cecal bacterial communities based on Bray–Curtis dissimilarity. PC1 and PC2 explained 18.1% and 11.7% of the variation, respectively. PERMANOVA indicated a significant overall difference in cecal bacterial (R^2^ = 0.3483, pseudo-F = 1.336, *p* = 0.015), whereas the overall PERMDISP test detected no significant difference in multivariate dispersion (F = 1.5221, *p* = 0.277). (**D**) Alpha diversity indices, including Good’s coverage, Observed species, Chao1, Shannon, and Simpson. Control: corn–soybean meal basal diet; T1: 10% palm kernel meal (PKM); T2: 12% rice bran meal (RBM); T3: 15% sunflower meal (SFM); T4: 8% *Zanthoxylum bungeanum* seeds (ZBS).

The rarefaction curves became progressively flatter as sequencing depth increased, suggesting that the sequencing coverage was sufficient to represent most bacterial taxa present in the samples (Figure 1A). Good’s coverage values were above 98% in all groups (Figure 1D), indicating adequate sequencing depth and reliable characterization of the cecal microbiota. A total of 4417, 4543, 3425, 4242, and 5302 amplicon sequence variants (ASVs) were detected in the control, T1, T2, T3, and T4 groups, respectively, of which 3789, 3915, 2797, 3614, and 4674 ASVs were unique to each group (Figure 1B). No significant differences were found between any treatment group and the control group in Chao1, Observed Species, Shannon, or Simpson indices (*p* > 0.05; Figure 1D). Principal coordinate analysis (PCoA) based on Bray–Curtis dissimilarity showed variation in cecal microbial community composition among treatments (Figure 1C). The first two principal coordinate axes (PC1 and PC2) explained 18.1% and 11.7% of the variation, respectively. PERMANOVA revealed a significant overall difference in cecal bacterial community composition among treatments (R^2^ = 0.3483, pseudo-F = 1.336, *p* = 0.015), whereas the overall PERMDISP test detected no significant difference in multivariate dispersion (F = 1.5221, *p* = 0.277).

**Figure 2:**
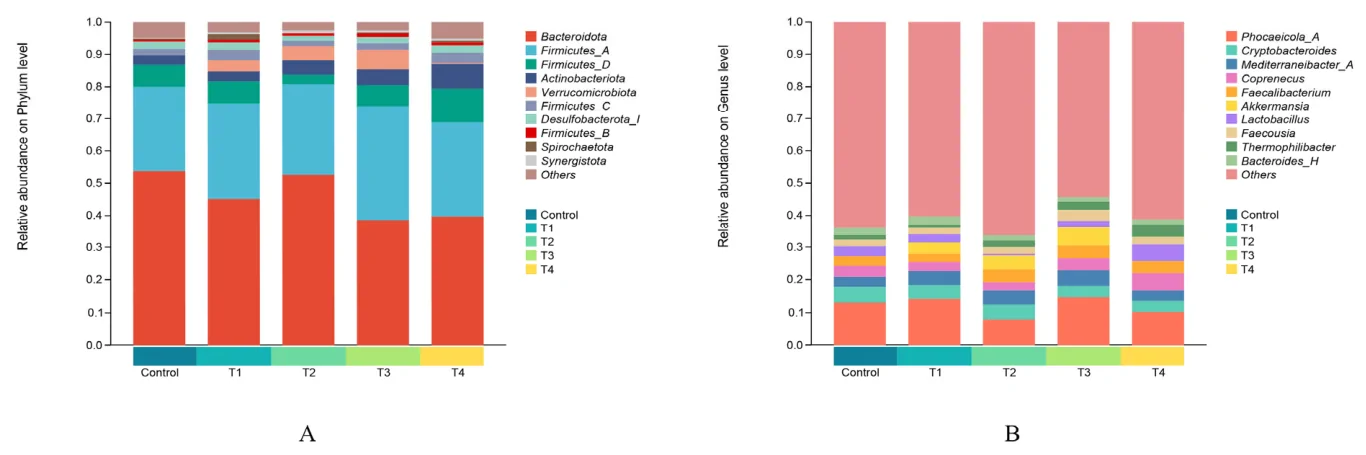
The 10 most abundant bacterial taxa at the phylum (**A**) and genus (**B**) levels in the cecal microbiota. Control: corn–soybean meal basal diet; T1: 10% PKM; T2: 12% RBM; T3: 15% SFM; T4: 8% ZBS.

The cecal bacterial community was dominated by *Bacteroidota*, *Firmicutes_A*, *Firmicutes_D*, *Actinobacteriota*, *Verrucomicrobiota*, *Firmicutes_C*, *Desulfobacterota_I*, *Firmicutes_B*, *Spirochaetota*, and *Synergistota* at the phylum level (Figure 2A). At the genus level, the predominant taxa included *Phocaeicola_A*, *Cryptobacteroides*, *Mediterraneibacter_A*, *Coprenecus*, *Faecalibacterium*, *Akkermansia*, *Lactobacillus*, *Faecousia*, *Thermophilibacter*, and *Bacteroides_H* (Figure 2B).

**Figure 3:**
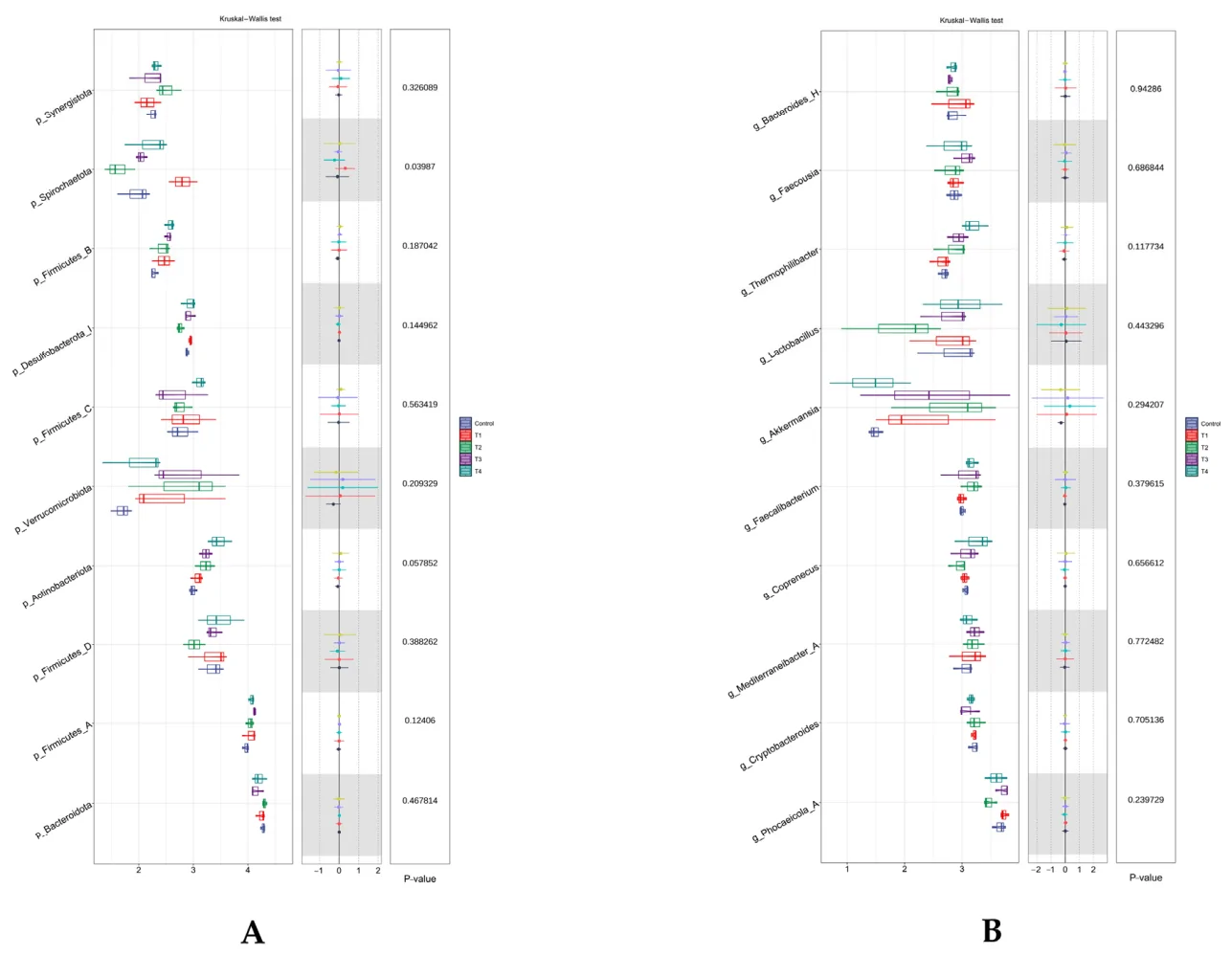
Comparison of the relative abundances of cecal bacterial taxa among the five dietary treatment groups in aged laying hens. (**A**) Phylum-level analysis; (**B**) genus-level analysis. Differences among groups were assessed using the Kruskal–Wallis test, with the Benjamini–Hochberg (BH) procedure applied to adjust for multiple testing. The figure displays unadjusted *p*-values, whereas BH correction was performed across all tested taxa at each taxonomic level. Control: corn–soybean meal basal diet; T1: 10% PKM; T2: 12% RBM; T3: 15% SFM; T4: 8% ZBS.

As shown in Figure 3, differences in the relative abundances of bacterial taxa among the five dietary treatment groups were evaluated using the Kruskal–Wallis test, followed by the Benjamini–Hochberg procedure to correct for multiple testing. At the phylum level, although *Spirochaetota* showed an unadjusted *p*-value below 0.05 (*p* = 0.0399), this difference was no longer statistically significant after correction (adjusted *p* = 0.4120). No significant differences were detected among the examined bacterial phyla after multiple-testing correction (adjusted *p* > 0.05). Similarly, at the genus level, none of the examined genera, including *Akkermansia*, *Faecalibacterium*, *Thermophilibacter*, and *Coprenecus*, exhibited significant differences among treatments after correction (adjusted *p* > 0.05).

### 3.3. Effects of Different Plant-Derived By-Products on Apparent Nutrient Utilization in Aged Laying Hens

Table 4 presents the apparent nutrient utilization of aged laying hens fed diets containing different plant-derived by-products. The T3 group exhibited significantly higher apparent utilization of all measured nutrients than the control group (*p* < 0.05). EE utilization was significantly higher in all treatment groups than in the control group (*p* < 0.05). Ca and ash utilization were significantly higher in the T2, T3, and T4 groups than in the control group (*p* < 0.05). In contrast, compared with the control group, DM utilization was significantly lower in the T1, T2, and T4 groups, TP utilization was significantly lower in the T1 group, and CP utilization was significantly lower in the T1 and T2 groups (*p* < 0.05).

### 3.4. Effects of Different Plant-Derived By-Products on Productive Performance and Egg Quality of Aged Laying Hens

As shown in Table 5, the laying rate was significantly higher in the T2 and T3 groups than in the control group (*p* < 0.05). No significant differences were observed in average egg weight, average daily feed intake, or feed-to-egg ratio among treatments (*p* > 0.05).

As shown in Table 6, eggshell thickness was significantly higher in all treatment groups than in the control group (*p* < 0.05). However, egg shape index, eggshell strength, egg weight, albumen height, yolk color, and Haugh units did not differ significantly among treatments (*p* > 0.05).

## 4. Discussion

This study investigated the effects of partial SBM replacement with four plant-derived by-products, including PKM, RBM, SFM, and ZBS, on digestive enzyme activities, cecal microbiota composition, apparent nutrient utilization, productive performance, and egg quality in aged laying hens.

### 4.1. Effects of Plant-Derived By-Products on Gastrointestinal Digestive Enzyme Activities in Aged Laying Hens

The digestion and utilization of dietary nutrients in laying hens are closely associated with the activities of endogenous digestive enzyme systems, which play essential roles in nutrient hydrolysis and gastrointestinal digestive processes [23]. Accordingly, digestive enzyme activity is widely regarded as an important indicator of gastrointestinal digestive function.

In the present study, dietary inclusion of different plant-derived by-products was associated with changes in gastrointestinal digestive enzyme activities, exhibiting clear segment-specific responses in the gizzard, duodenum, and jejunum. Notably, the PKM- and SFM-containing diets exhibited favorable responses in selected digestive enzyme activities, although these effects were not consistent across all gastrointestinal segments.

The positive response to PKM may be associated with its relatively high content of β-mannan-type non-starch polysaccharides. Previous studies have shown that dietary fiber components can modulate gastrointestinal development and chyme retention time, thereby influencing the secretion dynamics of digestive juices [10,24]. Moreover, it has been suggested that dietary fiber may participate in the adaptive regulation of pancreatic secretory function by modulating gastrointestinal activity and pancreatic responsiveness [23]. Therefore, the increased enzyme activity observed in the PKM group may be partially attributed to fiber-induced physiological adaptation of the gastrointestinal tract. However, because fiber fractions, gastrointestinal transit time, and pancreatic secretory function were not directly measured in the present study, this potential mechanism remains hypothetical.

Regarding SFM, its effects on digestive enzyme activities may be associated with its nutritional characteristics, including protein content, dietary fiber composition, and processing-dependent constituents. Previous studies have demonstrated that dietary SFM supplementation can improve intestinal morphological characteristics in aged laying hens, as evidenced by increased villus height-to-crypt depth ratio and goblet cell density, which may contribute to a more favorable environment for nutrient digestion and absorption [25]. In addition, performance-based studies have reported that partial replacement of SBM with SFM does not impair apparent nutrient utilization or overall laying performance in aged hens [26]. Collectively, these findings suggest that SFM can serve as a potential alternative feed ingredient when incorporated into appropriately formulated laying-hen diets. In the present study, the increases in selected digestive enzyme activities observed in the SFM group may therefore be associated with differences in dietary substrate composition and gastrointestinal adaptation to the SFM-containing diet.

The regulation of digestive enzyme activity by dietary fiber and plant-derived by-products is complex and is influenced by multiple factors, including inclusion level, fiber composition, and overall diet formulation [27]. In the present study, compared with the corn-SBM control group, the RBM and ZBS groups showed lower activities of certain digestive enzymes in specific gastrointestinal segments. However, digestive enzyme activity alone does not fully reflect overall digestive and absorptive capacity.

Overall, the four plant-derived by-products exerted distinct effects on digestive enzyme activities in aged laying hens, with the magnitude and direction of these responses varying among dietary treatments and gastrointestinal segments. These differences may be associated with variations in the nutritional characteristics of the ingredients and their dietary inclusion levels. Thus, the observed responses appeared to depend on the ingredient, enzyme, and gastrointestinal segment examined.

### 4.2. Effects of Plant-Derived By-Products on Cecal Microbiota in Aged Laying Hens

The gastrointestinal tract of animals represents a highly intricate environment, characterized by a dense, dynamically evolving, and dynamic microbial community [27]. The gut microbiota plays an integral role in maintaining host homeostasis, contributing to nutrient digestion and absorption, host immunity, and metabolism [28]. In poultry, the predominant microbial communities within the gut are primarily bacterial in nature [29], with the cecum identified as the principal site for microbial enrichment [30]. Protein, as a fundamental dietary component, demonstrates considerable variability in amino acid composition, utilization, and associated non-protein constituents—such as fiber type and content, fat composition, antinutritional factors, and bioactive substances—depending on its source. These compositional differences may influence the structure and metabolic potential of intestinal microbial communities. During the late laying period, changes in digestive physiology and nutrient utilization may increase the importance of evaluating alternative feed ingredients. Therefore, investigating cecal microbial communities can provide additional information on the responses of aged laying hens to dietary protein substitution. However, microbial taxonomic profiles alone cannot establish changes in microbial function or host health.

To assess species richness, the Observed Species Index and Chao-1 Index were employed, while the Shannon Index and Simpson Index were utilized to evaluate species diversity [31]. No significant differences in these indices were observed between any treatment group and the control group, indicating that partial replacement of soybean meal with PKM, RBM, SFM, or ZBS did not significantly alter cecal microbial alpha diversity under the experimental conditions. In contrast, principal coordinate analysis based on Bray–Curtis dissimilarity revealed differences in microbial community composition among treatments. This observation was supported by PERMANOVA (R^2^ = 0.3483, *p* = 0.015), while PERMDISP showed no significant differences in multivariate dispersion among groups (*p* = 0.277). These findings indicate that dietary protein substitution was associated with differences in overall cecal microbial community composition, despite the absence of significant changes in microbial richness and diversity. One possible explanation is that differences in the chemical composition of these feed ingredients, including their fiber fractions and other fermentable components, may have influenced microbial community assembly. However, the contribution of specific dietary components to the observed microbial differences was not directly investigated in this study.

Analysis of the cecal microbiota revealed that, at the phylum level, the dominant phyla in all diets containing non-conventional protein sources were Bacteroidota and several lineages of Firmicutes. This core community structure is highly consistent with the findings of several classic studies on poultry gut microbiota [32,33,34]. From a functional perspective, the Firmicutes and Bacteroidetes phyla synergistically participate in the body’s nutrient metabolism and form the key microbial foundation for maintaining intestinal digestive function [27,35]. The persistence of these major bacterial groups across dietary treatments suggests that the predominant taxonomic components of the cecal microbiota were broadly shared among groups. Although differences in the distribution of multiple taxa could potentially contribute to the observed beta-diversity pattern, this possibility was not directly established by the present analyses. Taxon-level comparisons using the Kruskal–Wallis test with Benjamini–Hochberg correction did not identify significant differences among treatments in the examined bacterial phyla (adjusted *p* > 0.05). Accordingly, the present results do not provide statistically significant evidence of treatment-related differences in the relative abundance of any of the examined phyla after correction for multiple testing. At the genus level, the cecal microbiota contained several bacterial genera potentially involved in intestinal microbial metabolism, including *Faecalibacterium*, *Akkermansia*, and *Thermophilicibacter*. Gül et al. (2025) [21] reported that adding SFM to the diet increases butyric acid concentration in the cecum of aged laying hens and improves their intestinal morphology. Certain members of *Faecalibacterium* are recognized for their capacity to produce butyrate, a microbial metabolite involved in intestinal epithelial energy metabolism. Similarly, studies on fermented rice bran supplementation in laying hens have reported increased butyric acid production and higher abundances of potentially beneficial bacteria [36]. Although fermented rice bran differs from the unfermented RBM used in the present study, these findings suggest that the effects of rice bran-derived ingredients on microbial fermentation warrant further investigation. In addition, Akkermansia has been associated with intestinal mucus metabolism and epithelial barrier function [37]. However, in the present study, no significant differences in the relative abundances of the examined bacterial genera were detected after Benjamini–Hochberg correction (adjusted *p* > 0.05). Therefore, although these genera have potentially relevant physiological functions, their contributions to the responses observed in the present study remain uncertain. Furthermore, Chen et al. [17] demonstrated that partial replacement of soybean meal with *Zanthoxylum bungeanum* seed meal (ZBM), a plant-derived by-product, improved nutrient utilization and several physiological indicators in broiler chickens, suggesting its potential as an alternative protein ingredient.

In the present study, the significant overall difference in microbial community composition, together with the absence of significant taxon-level differences after multiple-testing correction, suggests that dietary protein substitution may influence the collective structure of the cecal microbiota without producing statistically detectable differences in the relative abundances of individual bacterial taxa after multiple-testing correction. However, the microbiota analysis included only three biological replicates per treatment, and larger studies incorporating microbial functional measurements and metabolite profiling are needed to clarify the biological significance of these community-level differences. Overall, these findings suggest that partial replacement of soybean meal with different plant-derived by-products was associated with changes in the cecal microbial community composition of aged laying hens, with responses potentially varying according to the type of feed ingredient.

### 4.3. Effects of Plant-Derived By-Products on Apparent Nutrient Utilization in Aged Laying Hens

Apparent nutrient utilization is an important indicator for evaluating the nutritional value of feed ingredients and the extent to which dietary nutrients are utilized by poultry. Apparent nutrient utilization is influenced by a multitude of factors, including the composition of dietary fiber and antinutritional elements, feed processing techniques, the animal’s developmental stage, and intestinal health status. In the present study, the effects of the four plant-derived by-products on apparent nutrient utilization varied among nutrients and dietary treatments, with improvements in some parameters and reductions in others. Among the four treatments, the SFM treatment exhibited relatively favorable responses in apparent nutrient utilization, with significantly higher values for CP and certain mineral-related parameters than the control group. However, its responses were not consistently superior across all measured nutrients. SFM generally contains approximately 30–34% CP, although its protein content and amino acid profile may vary depending on the source and processing conditions. It is particularly abundant in functional amino acids such as arginine, glutamic acid, and aspartic acid [7]. The majority of prior research on SFM has focused on broiler chickens [38,39], leaving a paucity of systematic experimental data concerning its application in aged laying hens during the late laying phase. Although this study used broiler chickens as test subjects, research by Bilal et al. (2017) [40] confirmed that adding SFM to the diet does not reduce growth performance or apparent nutrient utilization in livestock and poultry. These findings support the potential use of SFM as an alternative protein ingredient in poultry diets, although its nutritional value should be evaluated in relation to the dietary formulation and inclusion level.

The responses to PKM, RBM, and ZBS also differed among the measured nutrients, suggesting that their nutritional characteristics and inclusion levels may jointly influence nutrient utilization. These variable responses are consistent with the recognition that the nutritional value of plant-derived feed ingredients depends on their chemical composition and dietary inclusion levels. Previous studies have reported that PKM can replace 25–75% of SBM in diets for aged laying hens without adversely affecting productive performance under the experimental conditions evaluated [41]. Choi et al. (2021) [11] reported that inclusion of 10% PKM in laying hen diets did not impair productive performance or feed efficiency, suggesting that PKM is a feasible alternative protein ingredient in laying hen diets. RBM has also been investigated as a potential alternative feed ingredient in poultry diets. Previous studies have reported that dietary inclusion of RBM at appropriate levels did not adversely affect laying performance or feed conversion efficiency, with favorable productive performance observed at inclusion levels of 10–15% [12]. Processing methods, including heat treatment and fermentation, may further modify its nutritional characteristics and improve its suitability for poultry diets [36]. The feed potential of ZBS is supported by previous research. Chen et al. (2024) [17] reported that partially replacing SBM with ZBS can improve nutrient utilization and physiological status in broiler chickens, supporting its potential as an alternative protein source. An important consideration in interpreting these findings is that PKM, RBM, SFM, and ZBS were evaluated at different dietary inclusion levels. Consequently, the observed differences reflect the combined effects of ingredient characteristics, inclusion levels, and overall dietary composition, rather than differences in intrinsic ingredient quality alone. The observed differences may reflect the combined effects of ingredient characteristics, inclusion levels, and the overall dietary formulations. Therefore, the present findings should not be interpreted as demonstrating general nutritional equivalence among PKM, RBM, SFM, ZBS, and SBM. Overall, the results indicate that partial replacement of SBM with these plant-derived by-products produced ingredient- and nutrient-specific responses in apparent nutrient utilization in aged laying hens. These findings suggest that the tested plant-derived by-products have potential for use in low-SBM diets under the experimental conditions evaluated. Further studies using comparable inclusion levels and controlled dietary compositions are needed to clarify their relative nutritional value.

### 4.4. Effects of Partial Replacement of Soybean Meal with Plant-Derived By-Products on Productive Performance and Egg Quality in Aged Laying Hens

Productive performance and egg quality are important indicators for evaluating nutrient utilization and physiological status in laying hens [42]. Productive performance reflects the ability of hens to utilize and convert dietary nutrients for egg production, whereas egg quality reflects aspects of the nutritional status, mineral deposition, and metabolic conditions involved in egg formation, as indicated by parameters such as eggshell strength, eggshell thickness, albumen quality, and yolk characteristics [43]. In the present study, partial replacement of SBM with PKM, RBM, SFM, or ZBS did not result in significant reductions in the measured productive performance parameters or major egg quality parameters in aged laying hens, suggesting that these plant-derived by-products could be incorporated into the experimental diets without adversely affecting these measured outcomes. However, the effects of different by-products varied, which may be associated with differences in their nutritional composition, nutrient availability, and potential effects on digestive function and intestinal microbial environments.

The inclusion of PKM resulted in a numerical increase in laying rate, a notable numerical decrease in feed-to-egg ratio, and a significant increase in eggshell thickness, while other productive performance and egg quality parameters were unaffected. Although PKM contains relatively high dietary fiber levels, it also provides residual protein, lipids, and fermentable carbohydrates. In the present study, although the PKM group exhibited reductions in the apparent utilization of certain nutrients, these changes were not accompanied by significant reductions in productive performance or the measured egg quality parameters. Adrizal et al. [44] reported that dietary supplementation with 15% and 30% PKM did not significantly affect laying rate, feed-to-egg ratio, egg weight, albumen height, Haugh units, or eggshell thickness in laying hens. In this study, the RBM group exhibited significantly higher laying rate and eggshell thickness than the control group, whereas average daily feed intake, egg weight, feed-to-egg ratio, and other egg quality parameters remained unchanged. Notably, the increased laying rate and eggshell thickness in the RBM group occurred alongside reductions in selected digestive enzyme activities and the apparent utilization of certain nutrients. However, the present study did not establish direct relationships between these digestive responses and productive performance. Samli et al. [45] reported that moderate inclusion of rice bran did not adversely affect productive performance or egg quality, whereas excessive supplementation reduced laying performance, indicating that the effects of RBM depend on inclusion level and dietary nutrient balance. Habibollahi et al. [46] found that dietary rice bran affected laying performance and egg weight at higher inclusion levels, whereas most egg quality parameters remained relatively stable; phytase supplementation further improved certain eggshell traits. In the present study, SFM supplementation significantly increased laying rate and eggshell thickness, whereas other productive performance and egg quality parameters were not significantly affected. The nutritional value of SFM is largely determined by its protein content, amino acid profile, and overall dietary nutrient balance. Shi et al. [47] and de Morais Oliveira et al. [48] reported that partial replacement of SBM with SFM under balanced amino acid conditions did not impair productive performance or egg quality in free-range laying hens. For ZBS, dietary inclusion of 8% ZBS did not significantly affect productive performance or major egg quality parameters, except for eggshell thickness, which was significantly increased. Furthermore, ZBS contains bioactive components, including unsaturated fatty acids, polyphenols, and flavonoids, which contribute to its distinctive nutritional composition. Chen et al. [17] reported that dietary replacement of SBM with ZBS improved nutrient utilization and growth performance in broilers, while Cao et al. [18] found that ZBS supplementation improved certain laying performance parameters without negatively affecting egg quality.

Overall, under the conditions of the present study, partial replacement of SBM with PKM, RBM, SFM, and ZBS did not result in significant reductions in the measured productive performance or egg quality parameters in aged laying hens during the late laying phase. Moreover, some treatments improved laying rate or eggshell thickness. Although the increases in eggshell thickness were relatively small, they may reflect modest changes in eggshell structural characteristics. However, these changes do not necessarily translate into improved eggshell strength or reduced breakage risk. Therefore, the biological and practical significance of the observed increases should be interpreted cautiously. The results of digestive enzyme activities, cecal microbiota composition, and apparent nutrient utilization further indicate that the tested plant-derived by-products affected several aspects of digestive physiology and intestinal microbial composition. However, the relationships between these responses and productive performance were not directly established in this study. Taken together, PKM, RBM, SFM, and ZBS showed potential as partial substitutes for SBM in diets for aged laying hens under the dietary formulations and inclusion levels evaluated in this study.

## 5. Conclusions

In conclusion, partial replacement of SBM with PKM, RBM, SFM, or ZBS produced ingredient- and gastrointestinal segment-specific responses in digestive enzyme activities in 80-week-old Hy-Line Brown laying hens. The alpha diversity of the cecal microbiota did not differ significantly among treatments, whereas overall microbial community composition differed significantly. Differences in the relative abundance of individual bacterial taxa were also observed, although these were not significant after multiple-testing correction. The SFM group showed significantly higher apparent utilization of all six measured nutrient parameters than the control group, whereas PKM, RBM, and ZBS exhibited mixed, nutrient-specific responses. RBM and SFM significantly increased laying rate, while all four treatments significantly increased eggshell thickness compared with the control group. Average egg weight, average daily feed intake, feed-to-egg ratio, and most other egg quality parameters were not significantly affected. Overall, PKM, RBM, SFM, and ZBS showed potential as partial substitutes for SBM under the specific dietary formulations and inclusion levels evaluated in this study, although their nutritional equivalence cannot be established from the present results. These findings provide experimental evidence for the potential application of plant-derived by-products as alternative feed resources in diets for aged laying hens. However, the limited number of birds used for microbial analysis, differences in dietary formulations, and the absence of direct measurements of microbial function limit the interpretation of the observed responses, and causal relationships between microbial changes and digestive function or productive performance cannot be established from the present findings. In addition, the evaluation of only one inclusion level per ingredient prevents the determination of the optimal inclusion levels. Furthermore, the present study did not comprehensively evaluate gut health. Future studies should investigate multiple inclusion levels of these plant-derived by-products, comprehensively characterize their nutritional composition, and conduct more robust gastrointestinal and microbiological assessments, including microbial functional analyses, to determine appropriate inclusion levels and further evaluate their potential application as alternative feed ingredients in laying hen diets.

## Figures and Tables

**Table 1:** Composition of the experimental diet. (%).

Ingredients	Experimental Group				
	Control	T1	T2	T3	T4
Corn	63.5	55.5	53.5	59	55.5
Soybean meal	22.5	19	19	11	21
Soybean oil	1	2	2	2	2
Stone powder	8	8.5	8.5	8.5	8.5
Premix ^1^	5	5	5	5	5
Palm kernel meal	0	10	0	0	0
Rice bran meal	0	0	12	0	0
Sunflower meal	0	0	0	15	0
*Zanthoxylum bungeanum* Seeds	0	0	0	0	8
Total	100				

^1^ The premix was included at 5% of the complete diet and contained the following nutrients per kilogram of premix: Cu, 300 mg; Fe, 2100 mg; Mn, 1100 mg; Zn, 1500 mg; vitamin K_3_, 16 mg; vitamin B_2_, 45 mg; folic acid, 12 mg; vitamin A (as acetate), 100,000 IU; vitamin D_3_, 40,000 IU; vitamin E, 160 IU.

**Table 2:** Nutrient composition of the experimental diets and alternative feed ingredients (as-fed basis).

Ingredients	Experimental Diets					Alternative Feed Ingredients			
	Control	T1	T2	T3	T4	PKM	RBM	SFM	ZBS
ME (MJ/kg)	11.18	11.18	11.20	11.19	11.21	10.09	9.0	7.9	9.49
CP (%)	14.86	14.90	14.84	14.89	14.91	16.13	16.11	32.29	11.58
TP (%)	0.41	0.42	0.40	0.41	0.41	0.67	1.50	0.92	0.21
Ca (%)	4.17	4.19	4.22	4.17	4.19	0.4	0.14	0.44	0.95
EE (%)	5.21	5.96	5.99	5.67	6.16	6.02	2.08	2.41	5.61
Ash (%)	14.67	13.97	14.98	14.12	14.02	10.72	10.64	6.40	5.89
Lys (%)	0.76	0.77	0.76	0.76	0.77	0.48	0.43	1.36	0.45
Met (%)	0.31	0.31	0.31	0.30	0.30	0.29	0.16	0.52	0.20
Met + Cys (%)	0.62	0.60	0.60	0.61	0.61	0.47	0.31	0.92	0.35
Thr (%)	0.56	0.57	0.56	0.56	0.57	0.46	0.41	1.15	0.27
Trp (%)	0.11	0.12	0.10	0.10	0.11	0.07	0.09	0.28	0.05
Leu (%)	1.29	1.29	1.28	1.27	1.30	1.01	0.95	2.26	0.62
Ile (%)	0.57	0.57	0.55	0.56	0.56	0.55	0.34	1.35	0.37
Val (%)	0.67	0.66	0.66	0.66	0.67	0.76	0.52	1.43	0.44
Arg (%)	0.95	0.96	0.97	0.95	0.96	1.68	0.52	2.77	0.72

Metabolizable energy (ME) was calculated according to NY/T 33-2004; Crude protein (CP), Calcium (Ca), Ether extract (EE), Crude ash (Ash), and Total phosphorus (TP) were analyzed, and amino acid contents were calculated. Lys, lysine; Met, methionine; Met + Cys, methionine plus cysteine; Thr, threonine; Trp, tryptophan; Leu, leucine; Ile, isoleucine; Val, valine; Arg, arginine. PKM, palm kernel meal; RBM, rice bran meal; SFM, sunflower meal; ZBS, *Zanthoxylum bungeanum* seeds.

**Table 3:** Activities of gastrointestinal digestive enzymes in aged laying hens fed diets containing different plant-derived by-products.

Items		Experimental Group					*p*-Value
		Control	T1	T2	T3	T4	
Gizzard	Pepsin (U/mgprot)	57.92 ± 8.63 ^b^	114.32 ± 34.14 ^a^	56.72 ± 21.51 ^b^	68.11 ± 20.72 ^b^	42.76 ± 15.41 ^b^	<0.01
Duodenum	Trypsin (U/mgprot)	2450.28 ± 194.44 ^ab^	2488.26 ± 210.34 ^a^	1999.53 ± 155.56 ^c^	2449.44 ± 142.53 ^ab^	2116.10 ± 313.72 ^bc^	0.013
	Chymotrypsin (U/mgprot)	20.67 ± 1.30 ^a^	25.20 ± 8.43 ^a^	12.38 ± 2.16 ^b^	23.03 ± 1.26 ^a^	13.80 ± 4.71 ^b^	<0.01
	Lipase (U/mgprot)	198.48 ± 19.12 ^a^	211.62 ± 20.19 ^a^	134.07 ± 19.25 ^bc^	176.37 ± 50.63 ^ab^	96.64 ± 37.21 ^c^	<0.01
	α-Amylase (U/gprot)	203.98 ± 7.04 ^a^	212.21 ± 2.01 ^a^	150.94 ± 9.52 ^c^	178.26 ± 28.37 ^b^	175.10 ± 13.30 ^b^	<0.01
Jejunum	Trypsin (U/mgprot)	2629.50 ± 263.21 ^c^	3161.79 ± 98.80 ^a^	2665.22 ± 175.65 ^bc^	2962.25 ± 114.25 ^ab^	2297.14 ± 267.62 ^d^	<0.01
	Chymotrypsin (U/mgprot)	22.94 ± 1.15 ^ab^	24.31 ± 2.80 ^a^	4.28 ± 1.07 ^d^	16.36 ± 4.36 ^c^	19.22 ± 3.09 ^bc^	<0.01
	Lipase (U/mgprot)	428.12 ± 243.64 ^ab^	306.01 ± 93.66 ^b^	252.53 ± 129.50 ^b^	639.40 ± 119.71 ^a^	261.98 ± 248.61 ^b^	0.041
	α-Amylase (U/gprot)	321.50 ± 3.19 ^c^	443.92 ± 5.54 ^a^	376.55 ± 2.74 ^b^	373.90 ± 6.80 ^b^	298.47 ± 1.44 ^d^	<0.01

Control: corn–soybean meal basal diet group, T1: palm kernel meal diet group, T2: rice bran meal diet group, T3: sunflower meal diet group, T4: *Zanthoxylum bungeanum* seed diet group. Values are presented as mean ± SD (*n* = 3 independent replicate units per treatment, with one hen sampled per replicate unit). Different superscript letters within the same row indicate significant differences among treatments (*p* < 0.05), as determined by one-way ANOVA followed by Duncan’s multiple range test.

**Table 4:** Effects of Different Plant-Derived By-Products on Apparent Nutrient Utilization in Aged Laying Hens (%).

Items	Experimental Group					*p*-Value
	Control	T1	T2	T3	T4	
EE	79.65 ± 0.71 ^c^	81.99 ± 0.32 ^b^	82.51 ± 0.43 ^b^	85.40 ± 0.09 ^a^	86.35 ± 0.15 ^a^	<0.01
DM	70.14 ± 0.02 ^b^	62.70 ± 0.01 ^d^	62.59 ± 0.10 ^e^	70.33 ± 0.01 ^a^	68.77 ± 0.03 ^c^	<0.01
Ca	44.36 ± 0.54 ^c^	44.25 ± 0.94 ^c^	51.14 ± 0.00 ^b^	54.42 ± 0.74 ^a^	54.00 ± 0.21 ^a^	<0.01
TP	39.76 ± 0.25 ^b^	30.95 ± 2.79 ^c^	39.39 ± 1.80 ^b^	44.69 ± 1.20 ^a^	39.98 ± 1.59 ^b^	<0.01
Ash	34.66 ± 0.27 ^b^	33.42 ± 1.99 ^b^	40.33 ± 2.96 ^a^	41.26 ± 1.06 ^a^	41.62 ± 3.00 ^a^	<0.01
CP	45.78 ± 0.38 ^b^	40.66 ± 0.30 ^c^	42.89 ± 0.90 ^c^	50.80 ± 0.74 ^a^	47.35 ± 2.88 ^b^	<0.01

EE, ether extract; DM, dry matter; Ca, calcium; TP, total phosphorus; CP, crude protein. Control: corn–soybean meal basal diet group, T1: palm kernel meal diet group, T2: rice bran meal diet group, T3: sunflower meal diet group, T4: *Zanthoxylum bungeanum* seed diet group. Values are presented as means ± SD. Different superscript letters within the same row indicate significant differences among treatments (*p* < 0.05), as determined by one-way ANOVA followed by Duncan’s multiple range test.

**Table 5:** Effects of Different Plant-Derived By-Products on Productive Performance of Aged Laying Hens.

Items	Experimental Group					*p*-Value
	Control	T1	T2	T3	T4	
Laying Rate (%)	80.74 ± 2.07 ^b^	81.11 ± 2.53 ^b^	82.75 ± 2.41 ^a^	82.33 ± 2.52 ^a^	81.75 ± 2.55 ^ab^	<0.01
Average egg weight (g)	63.94 ± 2.27	64.02 ± 2.08	63.02 ± 2.61	64.21 ± 2.16	63.88 ± 2.55	0.251
Average Daily Feed Intake(g)	115.81 ± 3.73	115.89 ± 2.06	115.60 ± 1.83	116.68 ± 4.61	114.92 ± 1.64	0.195
Feed-To-Egg Ratio	2.25 ± 0.09	2.23 ± 0.07	2.22 ± 0.06	2.21 ± 0.11	2.20 ± 0.09	0.244

T1: palm kernel meal diet group, T2: rice bran meal diet group, T3: sunflower meal diet group, T4: *Zanthoxylum bungeanum* seed diet group. Values are presented as means ± SD. Different superscript letters within the same row indicate significant differences among treatments (*p* < 0.05), as determined by one-way ANOVA followed by Duncan’s multiple range test.

**Table 6:** Effects of Different Plant-Derived By-Products on Egg Quality of Aged Laying Hens.

Items	Experimental Group					*p*-Value
	Control	T1	T2	T3	T4	
Egg shape index (%)	77.28 ± 2.04	76.97 ± 0.69	77.32 ± 1.13	76.42 ± 2.14	77.80 ± 3.37	0.836
Eggshell strength (N)	36.09 ± 0.90	36.36 ± 1.00	36.11 ± 0.75	37.15 ± 0.74	36.70 ± 0.73	0.173
Eggshell thickness (mm)	0.367 ± 0.006 ^b^	0.373 ± 0.002 ^a^	0.373 ± 0.003 ^a^	0.377 ± 0.006 ^a^	0.374 ± 0.002 ^a^	0.031
Egg weight (g)	60.09 ± 1.26	61.52 ± 1.45	61.57 ± 1.27	62.31 ± 1.16	61.59 ± 1.60	0.107
Albumen height (mm)	6.06 ± 0.29	6.47 ± 0.31	6.39 ± 0.39	6.57 ± 0.49	6.34 ± 0.37	0.216
Egg yolk color	5.17 ± 0.52	5.17 ± 0.41	5.00 ± 0.45	4.92 ± 0.49	5.00 ± 0.32	0.818
Haugh units	76.91 ± 3.33	79.21 ± 5.06	78.65 ± 5.20	79.70 ± 4.36	78.28 ± 5.37	0.658

T1: palm kernel meal diet group, T2: rice bran meal diet group, T3: sunflower meal diet group, T4: *Zanthoxylum bungeanum* seed diet group. Values are presented as means ± SD. Different superscript letters within the same row indicate significant differences among treatments (*p* < 0.05), as determined by one-way ANOVA followed by Duncan’s multiple range test.

## Data Availability

The corresponding author can provide access to the collected and analyzed data sets from this study upon request.

## Abbreviations

The following abbreviations are used in this manuscript:

SBM	Soybean meal
PKM	Palm kernel meal
RBM	Rice bran meal
SFM	Sunflower meal
ZBS	*Zanthoxylum bungeanum* seeds
Ca	Calcium
TP	Total phosphorus
CP	Crude protein
DM	Dry matter
EE	Ether extract
Ash	Crude ash
